# TRANSCRIPTOMIC SIGNATURES FOR AGING AND EXTREME OLD AGE IDENTIFIED IN THE LONG LIFE FAMILY STUDY

**DOI:** 10.1093/geroni/igad104.1969

**Published:** 2023-12-21

**Authors:** Mengze Li, Stefano Monti, Paola Sebastiani, Zeyuan Song, Tanya Karagiannis, Thomas Perls, Michael Brent

**Affiliations:** Boston University, Boston, Massachusetts, United States; Boston University, Boston, Massachusetts, United States; Tufts Medical Center, Boston, Massachusetts, United States; Boston University, Boston, Massachusetts, United States; Tufts Medical Center, Boston, Massachusetts, United States; Boston University, Boston, Massachusetts, United States; Washington University in St. Louis, St. Louis, Missouri, United States

## Abstract

Transcriptional studies of blood have previously identified changes in gene expression profiles associated with age. Centenarians, a rare population of individuals that reach 100 years of age, experience delays in aging-related diseases and mortality. Thus, the analysis of their blood transcriptome may help shed light on mechanisms contributing to healthy aging and extreme longevity, as well as enable the discovery of candidate molecular therapeutic targets. In this project, we analyzed RNAseq-based blood transcriptomic data and genetic data from 1,286 participants in the Long Life Family Study age ranged from 25 to 108. To identify transcripts with different abundances at different ages, we used linear mixed-effect models and included relevant covariates to control for genetic, socioeconomic, and technical confounders. We also estimated a genetic relationship matrix (GRM) from the genotype data to account for familial relatedness. We used the same models and covariates to compare transcripts’ abundance between the extreme old and younger participants to discover markers of extreme old age. Among the 11k RNA transcripts tested, we identified 5,249 aging markers (FDR ≤ 0.01) and 761 extreme old age markers (FDR ≤ 0.01). We successfully replicated our aging signature with previously published aging signature in another cohort with comparable Z-scores. Currently, further investigation is ongoing to distinguish markers that contribute to extreme longevity from markers that represent extreme old age. This study found large signatures of age and extreme old age and will serve as a valuable reference for future research to identify molecular mechanisms of healthy aging.

